# Supplementary material for: Multiplexed computations in retinal ganglion cells of a single type
Source: Nat Commun. 2017 Dec 6;8:1964. doi: 10.1038/s41467-017-02159-y (PMC5719075; doi:10.1038/s41467-017-02159-y)
Supplement: Supplementary file 1 — Supplementary information [file 41467_2017_2159_MOESM1_ESM.pdf]

Multiplexed computations in retinal ganglion cells of a single type: Supplemental Materials

Stephane Deny[1], Ulisse Ferrari[1], Emilie Mace[1,2], Pierre Yger[1],
Romain Caplette[1], Serge Picaud[1], Gašper Tkačik[3] and Olivier Marre[1]

October 18, 2017

[1]Institut de la Vision, INSERM UMRS 968, UPMC UM 80, CNRS UMR 7210, Paris.
[2]Present address: Neural Circuit Laboratories, Friedrich Miescher Institute for Biomedical Research, Maulbeerstrasse 66, 4058 Basel, Switzerland
[3]Institute of Science and Technology Austria, Klosterneuburg, Austria

Supplemental Figure 1: responses of OFF ganglion cells to a full field flash

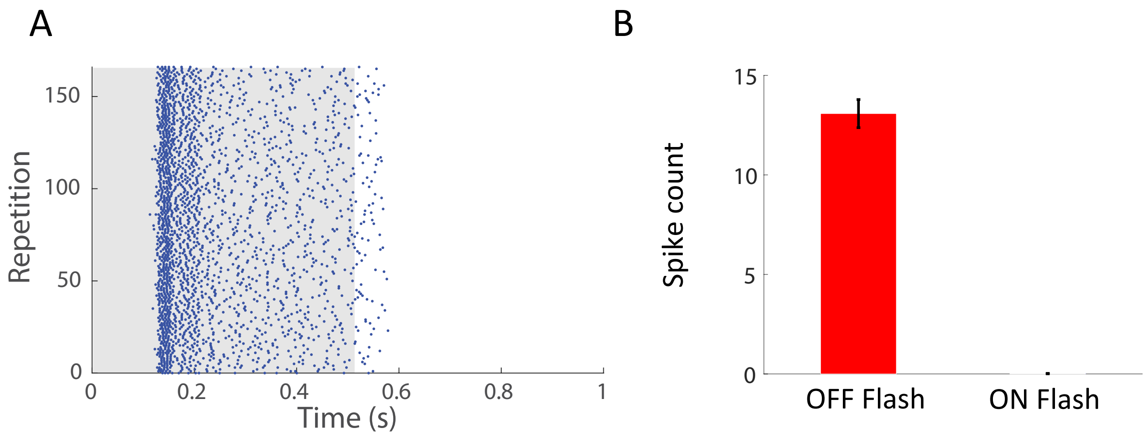

Supplemental Figure 1: **Responses of OFF ganglion cells to a full field flash A:** Raster of the response to a full field flash of one OFF ganglion cell of the type studied here. Gray area corresponds to the time window of the dark flash, white to the white flash. **B:** Average spike count in a time window from 100 ms after the flash to 500 ms after the flash, for OFF and ON flash. Error bars correspond to SEM (n=25 cells).

Supplemental Figure 2: responses of OFF ganglion cells to a randomly moving bar at different light levels

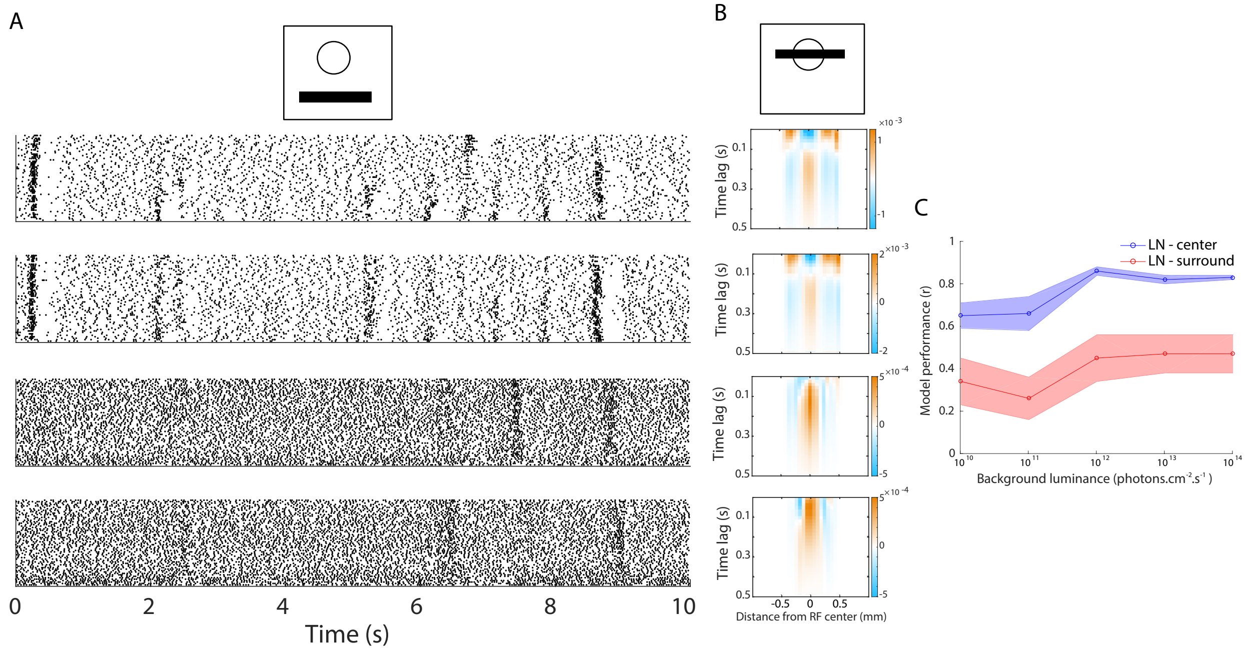

Supplemental Figure 2: **Responses of OFF ganglion cells to a randomly moving bar at different light levels** A: Raster of the response of one OFF ganglion cell to a randomly moving bar, for different background luminances. From top to bottom: 10^{14}, 10^{13}, 10^{11} and 10^{10} photons.cm^{-2}.s^{-1}. B: average linear filter for the LN model fitted to the data for each background luminance (n=6). C: average performance of the LN model for each level of luminance tested (n=6 for central cells ; n=7 for distant cells).

Supplemental Note 1: A subunit model to predict the responses to randomly moving textures

To test the generality of our model, we displayed a randomly moving texture composed of a random alternation of black and white bars over half of the visual field (see methods). Ganglion cells with their receptive field inside this half responded to the texture in a way that could be predicted by a LN model (supp. fig. 3A). However, cells far from the texture could not be predicted well (supp. fig. 3B). A subunit model similar to the one used for the moving bar (with slight differences for the subunit filters and non-linearities, see methods) could predict well these distant responses (supp. fig. 3C). This model architecture is therefore able to predict the responses to stimuli more complex than a bar.

Supplemental Figure 3: A subunit model to predict the responses to randomly moving textures

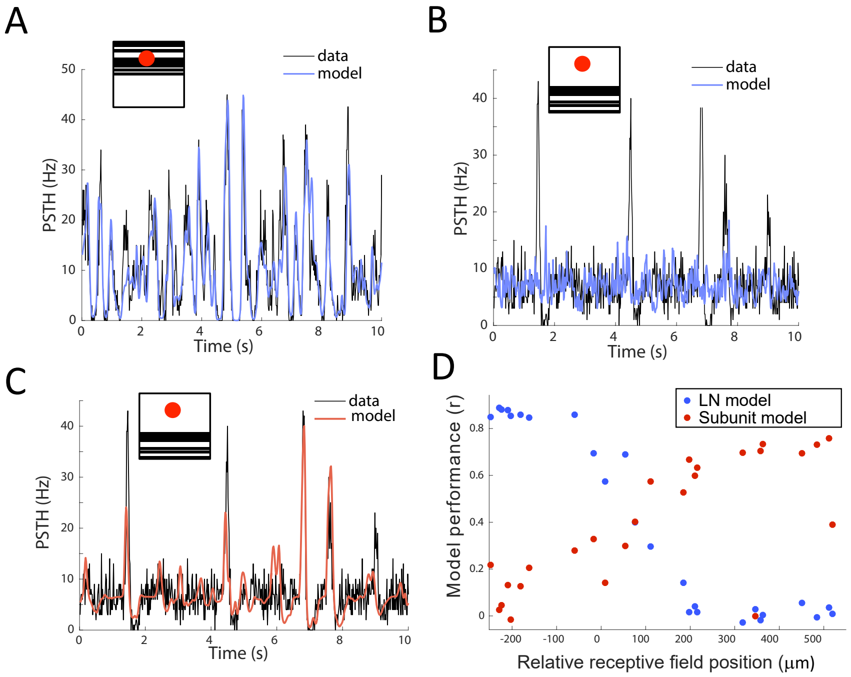

Supplemental Figure 3: **Responses of OFF ganglion cells to a randomly moving texture A:** Response (PSTH, black) of a ganglion cell whose receptive field center is stimulated by the texture, is predicted by the LN model (blue). $r = 0.87$. **B:** Response (PSTH, black) of the same ganglion cell when the texture is far from the receptive field center, is not predicted well by the LN model (blue). $r = 0.04$. **C:** Response (PSTH, black) of the same ganglion cell (as in B and C) to distant stimulation is predicted well by the subunit model (red). $r = 0.76$. **D:** Performance of the LN (blue) and subunit (red) models in predicting ganglion cell responses, as a function of the distance of the cell to texture border. Negative distances correspond to cells whose receptive field center is covered by the texture, while positive values correspond to the ones that are not covered by the texture. Note that here, in contrast with the case of the randomly moving bar, the performance of the subunit model decreased for central cells. This is because the subunit non-linearity employed here (see methods) did not allow this model to approximate a quasilinear behaviour with a combination of subunits.

Supplemental Figure 4: Suppression of distant responses by a bar moving inside the receptive field center in ON ganglion cells

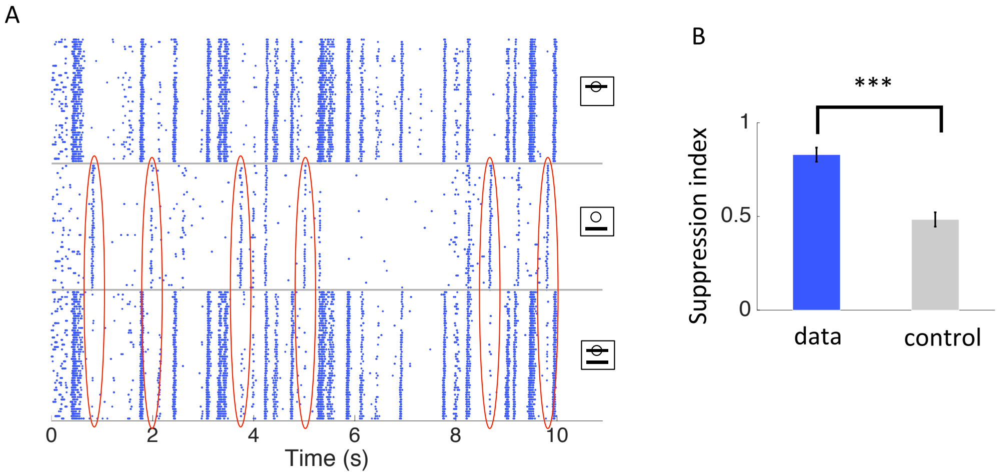

Supplemental Figure 4: **Suppression of distant responses by a bar moving inside the receptive field center in ON ganglion cells. A:** Response raster of a single cell to a moving bar presented in and/or outside of its receptive field center. Each dot is a single spike from the recorded cell. Each line corresponds to a different repetition of the same stimulus. top: bar moving inside the receptive field center. middle: bar moving outside the receptive field center. bottom: both bars displayed together. Red ellipses indicate examples where the response to the distant bar is strongly suppressed by the stimulus inside the receptive field center. **B:** Suppression index for real cells (data, blue) and due to noise (control, gray); see methods for details. Data are represented as mean ± SEM (n = 12 cells). The three stars indicate that the p-value of a two-sample t-test was lower than 0.001.

Supplemental Figure 5: subunit weight difference

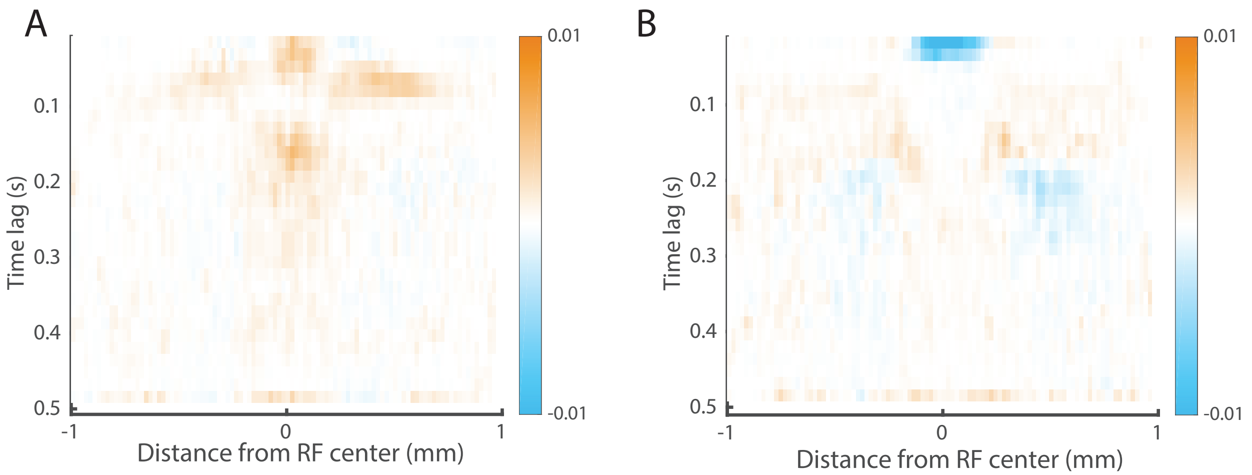

Supplemental Figure 5: **A:** ON subunit weight difference between the single distant bar stimulus and the combined bar stimulus. This corresponds to the difference between fig. 4D and C. **B:** Same as **A** for OFF-subunit weights.

Supplemental Note 2: linear summation followed by non-linearity cannot explain the suppression observed

A possible model to explain the suppression of the response to a distant bar by displaying a central bar is that the subunit outputs from the central bar and from the distant bar are all summed linearly in a global signal. This signal is then passed through the non-linearity to predict the firing rate. In this hypothesis, the response to the distant bar is suppressed because, at the same time, the central bar triggers a negative signal that cancels the activation due to the distant bar. We tested if this model of a linear summation of inputs followed by a global nonlinearity could predict the responses to the two bars, and found that it did not perform well at predicting the response to the two bars displayed simultaneously, compared to a model fitted directly on the responses to the two bars (supp. fig. 6D). Moreover, it largely underestimated the suppression index (see Eqs.12-13 in methods and supp. fig. 6E). We found similar results for the responses to the texture (supp. fig. 6A,B,C).

In particular, a consequence of this linear summation hypothesis is that a spiking response triggered by the distant stimulus can only be suppressed if the central stimulus triggers, at the same time, a negative signal. As a result, the distant response should not be suppressed if, at the same time, the central stimulus triggers even a moderate level of spiking activity, which indicates that there is no negative signal at that time. In the case of the texture, we found clear examples where, despite a moderate level of spiking activity triggered by the central stimulus, the response to the distant stimulus was still suppressed when the two stimuli were presented simultaneously (supp. fig. 6A). To quantify this we measured the suppression index only during periods where there was a minimal amount of spiking activity triggered by the central stimulus. We found that there was still a high suppression index in that case (supp. fig. 6F). We concluded that the linear summation model could not account for the observed suppression.

We show here that the suppression index is mostly independent of the firing rate. Note that this observation is not incompatible with the gain control mechanism of suppression presented in fig. 5 D,E. In the present case, the firing rate is estimated on the present time bin of 16 ms, while the gain control acts with a much larger time constant.

Supplemental Figure 6: linear summation followed by non-linearity cannot explain the suppression observed

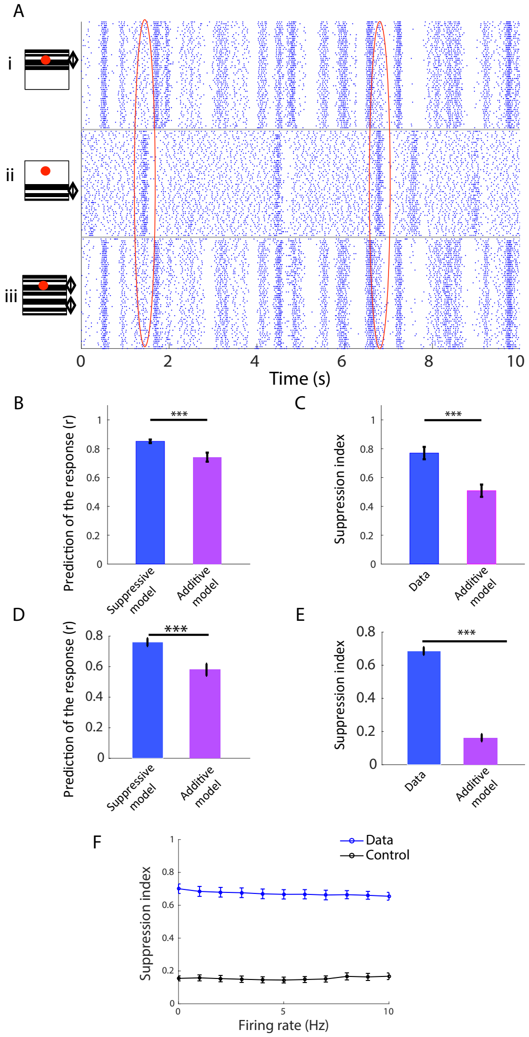

Supplemental Figure 6: **A:** Response raster of a single cell to a randomly moving texture presented in and/or outside of its receptive field center. Each dot is a single spike from the recorded cell. Each line corresponds to a different repetition of the same stimulus. i: texture moving inside the receptive field center. ii: texture moving outside the receptive field center. iii: both textures displayed together. Red ellipses indicate examples where the response to the distant texture is strongly suppressed by the stimulus inside the receptive field center, while there was a moderate spiking response to the central texture alone. **B:** Performance of two models in predicting the responses to the two randomly moving textures. In the suppressive model, the model is fitted on the responses to the two textures. In the additive model, the prediction signal (before the last non-linearity) from the model fitted to the central texture responses, and the prediction from the model fitted to the distant texture responses, are summed and then passed through the non-linearity. Data are represented as mean ± SEM (n=12). Performance is significantly lower for the additive model (The three stars indicate that the p-value of a paired-sample t-test was lower than 0.001). **C:** Suppression index estimated on the data, or on the additive model described in B. Performance is significantly lower for the additive model ($p \leq 10^{-3}$, paired-sample t-test). Data are represented as mean ± SEM (n=12). **D:** Same as B, but for the bar stimuli ($p \leq 10^{-3}$, paired-sample t-test). **E:** Same as C, but for the bar stimuli ($p \leq 10^{-3}$, paired-sample t-test). **F:** Suppression index for the data and due to noise (see methods) estimated on cells responding to the texture stimuli, taking into account only the time bins with an average firing rate in response to the central texture above a threshold firing rate (x-axis). Data are represented as mean ± SEM.

Supplemental Note 3: Sensitivity of the model to changes in the absolute position

Our results show that cells close to the bar were much more sensitive to the bar position than distant cells. Here we show that the subunit model fitted on the cell responses also had this property. For this we directly used our model to estimate the amount of information about a change in the absolute position of the bar trajectory.

To determine the sensitivity of each cell to a change in the absolute position we estimated the Kullback-Leibler divergence $d_{\mathrm{KL}}(\Delta x)$ between the cell response to an initial trajectory $x(t)$, and the response to the same trajectory displaced by a small constant shift Δx. We picked randomly a time T in the stimulus trajectory, and extracted the trajectory $x(t)$ of the bar for t between T and $T + DT$ (in the following $DT = 16$ s but the exact value did not change significantly the results). For each cell we then estimated $d_{\mathrm{KL}}(\Delta x)$ between the model response to $x(t)$ and the model response to $x(t)+\Delta x(t)$, where $\Delta x(t) = \text{constant}$ is a uniform perturbation of the trajectory, for $t \in [T, T + DT]$. We repeated this estimation many times for different times T (each point in the scatter plots of supp. fig. 2 corresponds to one cell and one choice of T).

To estimate $d_{\mathrm{KL}}(\Delta x)$ we assumed that Δx is small, so that we can expand the Kullback-Leibler divergence up to the second order to obtain:

$$d_{\mathrm{KL}}(\Delta x) \approx \frac{1}{2} \sum_{t,t' \in [T, T+DT]} \Delta x(t) I_{t,t'} \Delta x(t') \tag{1}$$

where the matrix $I_{t,t'}$ is the Fisher Information Matrix of the response distribution conditioned to the stimulus:

$$I_{t,t'} = \sum_{\tau = T \ldots T+DT+L} \frac{1}{r(\tau)} \frac{\partial r(\tau)}{\partial x(t)} \frac{\partial r(\tau)}{\partial x(t')} \tag{2}$$

where $x(t)$ is the position at time t and $r(\tau)$ is the firing rate at time τ predicted by the subunit model in response to the stimulus. $L = 0.5$ s corresponds to the maximal latency of the response to the stimulus. We then defined the sensitivity as $d_{\mathrm{KL}}(\Delta x)$ for a normalized perturbation such that $\sum_t \Delta x(t)^2 = 1$. We estimated this quantity for all the cells where the model had a very good prediction performance ($r \geq 0.7$ in fig. 2F).

For cells close to the bar, sensitivity to changes in the absolute position of the bar was high and strongly decreased for distant cells (supp. fig. 6A). We then asked if this decrease is specific to this uniform perturbation, or if it is a global decrease of sensitivity of distant cells to any perturbation. To test this we estimated the maximal sensitivity of each cell, which is the largest eigenvalue of the Fisher information matrix $I_{t,t'}$. We normalized the previous sensitivity values by this maximal sensitivity to obtain a "normalized sensitivity". Even after this, we observed a decrease of this normalized sensitivity with distance (supp. fig. 6B). These results show that the model fitted on the cells has the same property as found on the data previously: central cells were much more sensitive to stimulus position than distant cells.

Supplemental Figure 7: Sensitivity of the model to changes in the absolute position

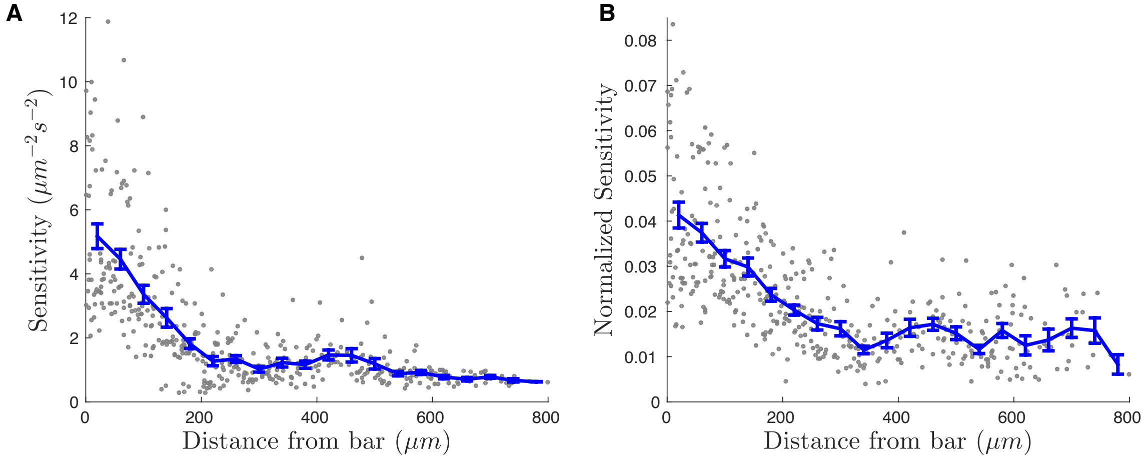

Supplemental Figure 7: **A:** Sensitivity (see text for definition) of cells to a change in the absolute position of the stimulus as a function of the distance of the cell to the bar. Each point corresponds to one cell and one choice of T (see text). **B:** Normalized sensitivity (see text for definition) of cells to a change in the absolute position of the stimulus as a function of the distance of the cell to the bar.

Supplemental Figure 8: analysis of the subunit model

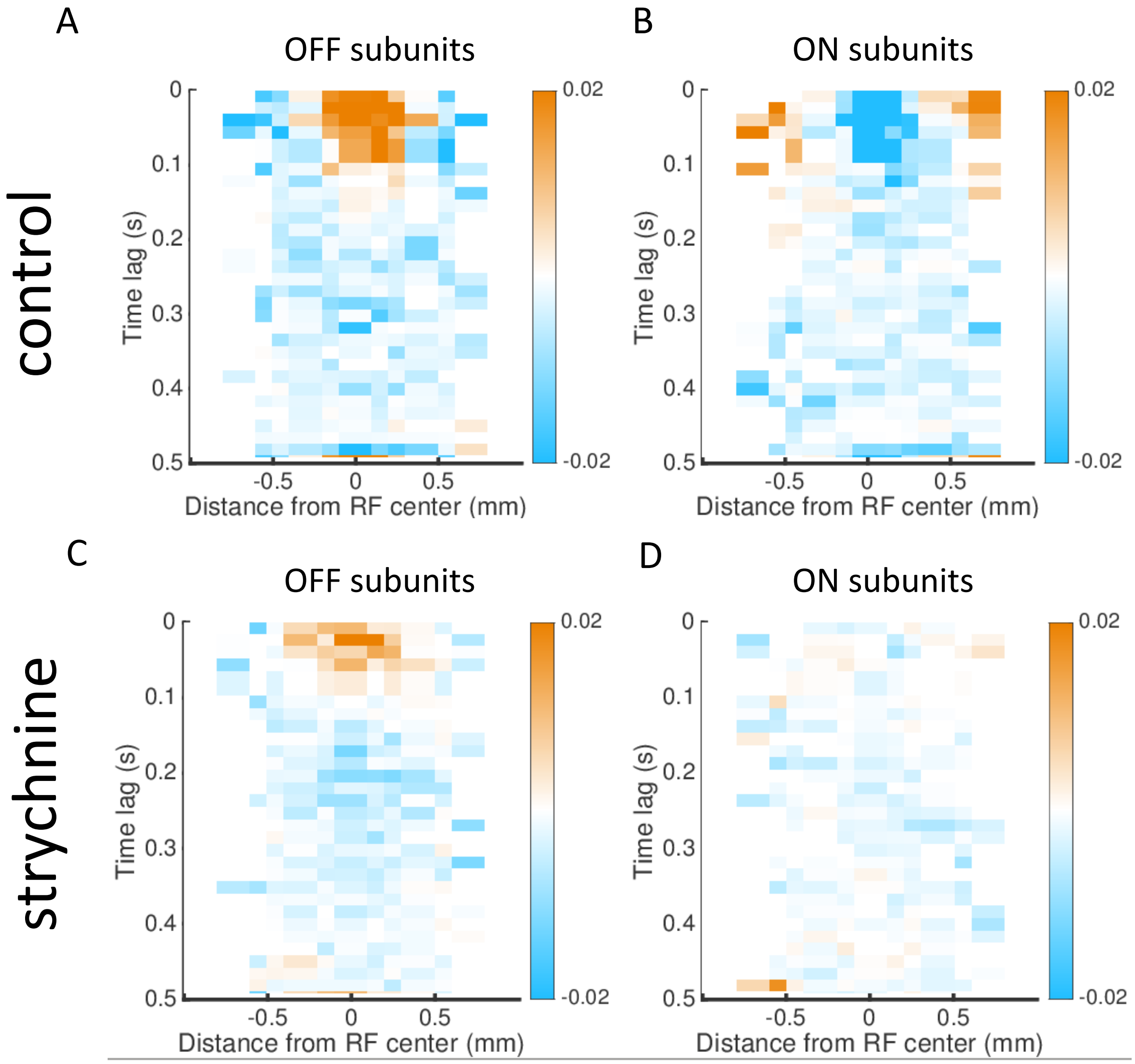

Supplemental Figure 8: Average filters of the subunit model in response to a central bar before (A, B) and after (C,D) adding strychnine, for OFF (A, C) and ON (B, D) subunits. The central ON subunits and the classical OFF inhibitory surround are suppressed by strychnine. Note that these changes are compatible with the preservation of the response to a bar flashed in the center that we observed above. The flashed bar elicits only OFF responses and thus should not be affected by the disappearance of ON negative weights in the center. Moreover, the OFF negative weights are not present in the center but only in the surround, so their disappearance should not affect the response either.

Supplemental Figure 9: distant responses suppressed by LAP-4

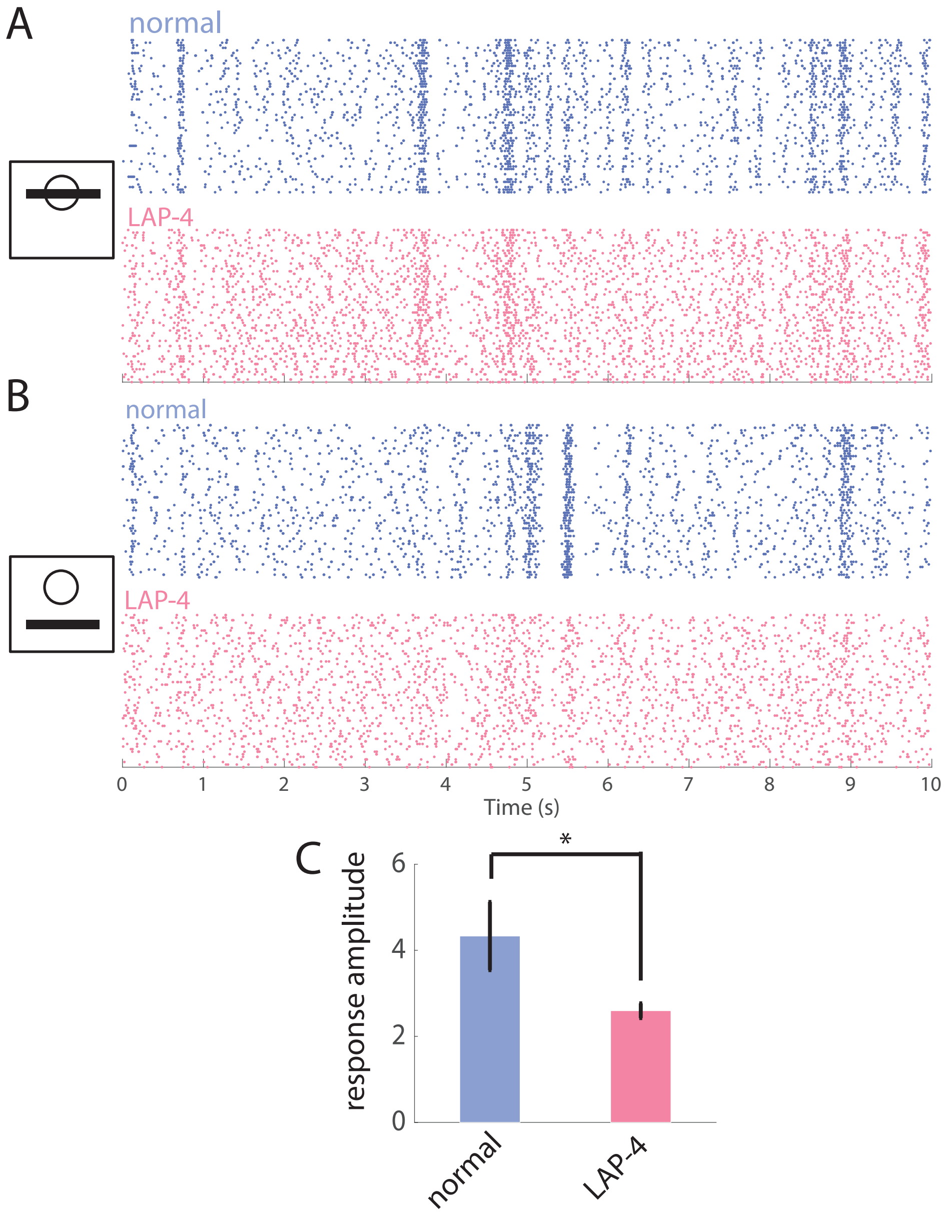

Supplemental Figure 9: **A:** Raster of an OFF cell responding to a repeated sequence of random motion in the center of its receptive field, before and after adding LAP-4 to the bath **B:** Raster of an OFF cell in response to the same stimulus far away from its receptive field (central axis of the bar trajectory 300 microns away from the receptive field center of the cell), before (blue) and after (pink) adding LAP-4 to the bath. **C:** Distant response amplitude (see methods) for normal (blue) and LAP-4 (pink) condition (n=16 cells). The star indicates that the p-value of a two-sample t-test was lower than 0.05.
